# The Automobile

**Published:** 1901-12

**Authors:** 


### The Automobile.

WITH the addition of the motor vehicle to our methods of transit, new problems of danger are presented to pedestrians in particular in every large city. Already in Buffalo a sufficient number of accidents has occurred, attributable to carelessness by chauffeurs, or the too great speed of their machines, to justify some supervision of this new method of travel and traffic.

With the increase in trolley cars along our principal streets, and the large army of male and female bicyclists, together with the reckless delivery wagon drivers, it has become nearly impossible for people afoot to cross without danger to life or limb. Now, to all this is added the automobile with electric or steam-power, driven by a novice or other indifferent person who cares for naught except his own comfort or sport.

Most persons who preside at the lever of these carriages appear to regard their whole duty as discharged when they have clanged their bells which, for the most part, is done at a time when they startle a person or a horse and add confusion to the already confounded pedestrian or beast. The steam vehicles, too, are a source of fear to many horses which is a further cause of danger.

We are fully of the opinion that the automobile is a useful carriage and will grow in favor if it is properly handled; but those who use it must understand that they do not possess the right of way over every other method of traffic. They must be made to realise that as yet they are in minority and cannot trample on the rights of others with impunity, especially when they use such a powerful machine fraught with danger, if uncivilly or unskilfully guided.

We have felt impelled to allude to this matter now in the hope that some action may be taken to properly regulate this useful carriage, and prevent accidents that belong to the preventable class.

